# Tuning the buried interface with d-sorbitol-modified mixed SAMs for high-efficiency inverted perovskite solar cells

**DOI:** 10.1039/d6ra01990k

**Published:** 2026-04-17

**Authors:** Adem Mutlu, Necip Ali Tuna, Destan Toksoz, Cem Tozlu

**Affiliations:** a Ege University, Solar Energy Institute 35100 Izmir Turkiye adem.mutlu@ege.edu.tr destantoksoz2000@gmail.com; b Department of Materials Science and Engineering, Izmir Katip Celebi University Izmir Turkiye necipalituna@gmail.com; c Graphene Application and Research Center, Izmir Katip Celebi University Cigli 35620 Izmir Turkiye cem.tozlu@ikcu.edu.tr

## Abstract

One of the key bottlenecks limiting high efficiency and long-term stability in perovskite solar cells (PSCs) is non-radiative recombination and slow interfacial-process-related losses at the buried transparent conducting oxide/perovskite interface. Here, a mixed 2PACz/MeO-2PACz self-assembled monolayer (SAM) (1 : 3, w/w) is deposited on high-haze FTO to form a hole-selective contact in an inverted (p–i–n) architecture, and the buried interface is further modified with d-sorbitol (DS), a polyol bearing multiple –OH groups. X-ray diffraction confirms that mixed-SAM formation and DS treatment preserve the perovskite crystal structure without detectable secondary phases, while sharpening diffraction features consistent with more controlled crystallization. The mixed-SAM enables smoother perovskite growth, and DS treatment preserves this smooth morphology while increasing surface wettability. Steady-state photoluminescence increases and time-resolved PL exhibits prolonged decay components after DS treatment, consistent with reduced non-radiative recombination losses associated with the buried interface. Electrochemical impedance spectroscopy shows an increased recombination resistance (*R*_rec_), supporting reduced interfacial recombination. Surface-sensitive characterization further indicates DS-assisted reorganization of the mixed-SAM/FTO contact toward a more uniform and chemically accessible anchoring configuration. Consequently, DS-modified mixed-SAM device increases *J*_sc_ from 19.3 to 22.4 mA cm^−2^ and *V*_oc_ from 1060 to 1080 mV, boosting PCE from 14.1% to 16.1%, while lowering the hysteresis index from 0.032 to 0.022. Additionally, DS-treated devices exhibit improved inert shelf-storage stability, retaining 81.7% of the initial normalized PCE after 8 days, *versus* 42.2% for reference. Overall, polyol-assisted mixed-SAM engineering provides a simple, solution-processable route to mitigate slow interfacial losses and improve p–i–n PSC performance and stability.

## Abstract 2

One of the key bottlenecks limiting high efficiency and long-term stability in perovskite solar cells (PSCs) is non-radiative recombination and slow interfacial-process-related losses at the buried transparent conducting oxide/perovskite interface.

## Graphical abstract

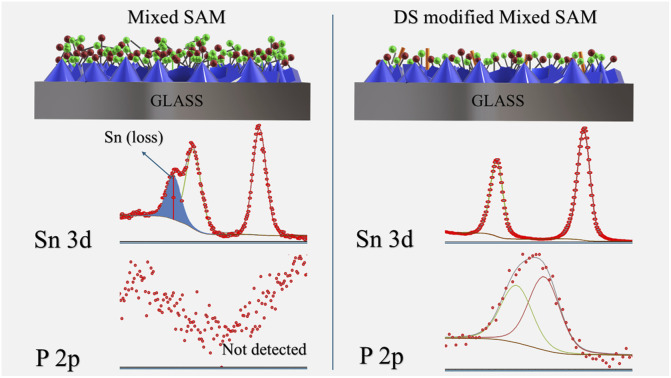

## Introduction

Perovskite solar cells (PSCs) have become one of the fastest-advancing photovoltaic technologies over the past decade, owing to their high absorption coefficients, bandgap tunability, long carrier diffusion lengths, and compatibility with low-temperature, solution-based processing.^1–3^ Despite rapid progress, transferring PSC performance from small-area laboratory devices to scalable modules while maintaining long-term operational stability remains challenging, largely due to interfacial non-radiative recombination, ion-migration-driven hysteresis, energy-level misalignment, and limited reproducibility in film formation.^4^ In particular, the “buried interface” between the transparent conducting oxide (TCO; fluorine doped tin oxide (FTO)/indium tin oxide (ITO)) and the perovskite constitutes a major recombination hotspot that governs charge-extraction efficiency and constrains both the open-circuit voltage (*V*_oc_) and the fill factor (FF).^5^ Accordingly, interfacial engineering has emerged as a central research strategy for efficiency stability optimization in PSC architectures.^6,7^

In conventional PSC architectures, organic/inorganic hole-transport layers (HTLs) can provide favorable energy alignment and selectivity; however, they also introduce additional processing steps and material costs, may suffer from thermal/chemical instability, and can create new defect-mediated loss channels at interfaces.^8,9^ In this context, self-assembled monolayers (SAMs) offer a compelling alternative by chemically functionalizing the TCO surface to tune its work function, improving energy-level alignment *via* interfacial dipoles, and enabling selective-contact behavior without increasing series resistance owing to their ultrathin nature.^10^ Among them, carbazole-based phosphonic-acid SAMs (*e.g.*, [2-(9*H*-carbazol-9-yl)ethyl]phosphonic acid (2PACz) and [2-(3,6-dimethoxy-9*H*-carbazol-9-yl)ethyl]phosphonic acid (MeO-2PACz)) have been widely adopted in high-performance inverted PSCs due to their low-temperature processability and robust binding to TCO surfaces.^11–14^ However, the fixed dipole moment and surface energy associated with a single SAM species may not always provide the optimal wettability and crystallization environment for diverse perovskite compositions and precursor formulations, leading to pronounced variations in film morphology, grain-boundary and interfacial defect distributions.^15^ Recently, “mixed SAM” strategies where two different SAM molecules are blended in controlled ratios have attracted considerable attention as an effective route to overcome this limitation by enabling more refined control over both work function/dipole tuning and surface energy.^16–18^ The mixing approach can regulate precursor spreading and coating dynamics, thereby promoting more compact film growth; concurrently, it can optimize interfacial chemical bonding and electrical selectivity, reducing non-radiative recombination.^19,20^ While mixed SAMs have proven effective in regulating buried-contact energetics and suppressing interfacial recombination, how mixed SAMs influence interfacial ionic processes (*e.g.*, ion accumulation, dipole reorientation, and halide-vacancy-related trap states) and how these effects translate into device performance through specific mechanisms still being actively investigated.^21,22^ Recent studies further highlight that defect-rich interfacial chemistries can concurrently amplify non-radiative recombination and facilitate ionic migration/accumulation, thereby accelerating performance decay under bias and during aging.^23^ Accordingly, hydroxyl-rich and polar chemical environments introduced by post-treatments have been shown to mitigate defect-mediated loss channels and prolong carrier lifetimes, underscoring the utility of “soft” chemical regulation for stabilizing interfacial ionic–electronic dynamics. Moreover, compared with rigid oxide interlayers (*e.g.*, Al_2_O_3_) that can introduce an additional dielectric barrier and require thickness control, polyol-based molecular modifiers offer a “soft”, solution-processable handle to tune the buried SAM/perovskite interface *via* polar interactions and hydrogen bonding without necessarily penalizing charge extraction.^24–26^ In parallel with these developments, mixed-SAM approaches have been pursued *via* different molecular architectures and interfacial-engineering strategies. For example, a double-layer carbazole–phosphonic-acid strategy has been shown to direct bottom-up crystallization and suppress halide segregation in wide-bandgap perovskites, primarily by regulating nucleation and composition uniformity at the buried contact.^27^ In a separate direction, mixed-SAM approaches combined with polymeric interlayers have been utilized to improve charge collection and fill factor, particularly in thick-absorber devices where transport losses and series-resistance effects become prominent.^28^ However, how a mixed-SAM interface can be post-tuned *via* a soft, hydroxyl-rich molecular treatment to directly regulate buried-contact ionic–electronic dynamics, particularly on textured high-haze FTO electrodes, remains underexplored. In this regard, polyol-assisted processing offers a low-temperature, solution-processable route to reconfigure the mixed-SAM/oxide interfacial environment through d-sorbitol (DS) incorporation, *via* polar interactions and likely hydrogen bonding. This interfacial strategy reduces interface trap-state density, enhances wettability, and improves the accessibility of anchoring sites at the buried interface.

In this study, we investigate a mixed 2PACz/MeO-2PACz SAM (1 : 3, w/w) as a hole-selective interface on high haze FTO and further modify the mixed-SAM surface with DS, a polyol bearing multiple –OH functionalities.^29^ The impact of this strategy is systematically elucidated by correlating surface energetics, interfacial morphology, and charge-carrier dynamics across the buried contact. Water contact-angle measurements verify that DS increases the wettability of the mixed SAM on FTO, consistent with enhanced surface polarity that can promote more uniform precursor spreading. Beyond the perovskite overlayer, atomic force microscopy (AFM) topography and phase mapping on FTO (including high-haze and low-haze substrates) reveal that the mixed-SAM is highly sensitive to the rinsing protocol and that DS-assisted processing induces the most pronounced reorganization on textured high-haze electrodes, providing strong evidence that the treatment modifies the buried-contact prior to perovskite deposition. Complementary X-ray photoelectron spectroscopy (XPS) further supports DS-assisted interfacial reconfiguration: a clear P 2p signal is resolved only after DS treatment, whereas P 2p remains below the detection limit in the mixed-SAM reference across multiple measurement spots, accompanied by a distinct Sn (loss) contribution, together indicating improved chemical accessibility and uniformity of the SAM anchoring configuration at the high haze FTO surface. Mixed-SAM formation followed by DS polyol modification at the buried FTO/SAM/perovskite junction reduces non-radiative recombination losses, as evidenced by an enhanced steady-state photoluminescence (PL) response and systematically prolonged time-resolved PL (TRPL) lifetimes. Electrochemical impedance spectroscopy (EIS) relates these photophysical trends to device level recombination by extracting an apparent recombination resistance from equivalent-circuit analysis. Overall, the results support a coherent picture in which DS polyol treatment of the mixed SAM surface tunes the surface energy and coating kinetics while preserving the smooth morphology enabled by the mixed-SAM. This interfacial regulation reduces non-radiative recombination losses at the buried contact, mitigating slow interfacial relaxation processes, and improves stability during inert shelf storage, collectively yielding reproducible performance gains with reduced hysteresis.

## Experimental methods

### Materials

Fluorine-doped tin oxide (FTO)-coated glass substrates were used as transparent electrodes. Unless otherwise stated, high-haze FTO (2.2 mm glass thickness; light transmittance 80–82%; sheet resistance 7 Ω per sq, haze: 11%) was employed. For comparison, low-haze FTO substrates (TEC-15, haze: 2.5%) were also used. Commercial 2PACz and MeO-2PACz were purchased from TCI. For perovskite precursors, formamidinium iodide (FAI) was purchased from Solaveni, methylammonium bromide (MABr) and cesium iodide (CsI) from Sigma-Aldrich, lead(ii) iodide (PbI_2_) from TCI, and lead(ii) bromide (PbBr_2_) from Sigma-Aldrich. [6,6]-Phenyl-C_61_-butyric acid methyl ester (PCBM) was obtained from Lumtec as the electron-transport layer, and bathocuproine (BCP) from Sigma-Aldrich was used as an interlayer. Anhydrous chlorobenzene was used as an anti-solvent. All solvents (anhydrous dimethyl formamide (DMF), anhydrous dimethyl sulfoxide (DMSO), anhydrous chlorobenzene, and anhydrous isopropyl alcohol) were purchased from Sigma-Aldrich.

### Characterization

Current density–voltage (*J*–*V*) measurements were performed under an AM 1.5G solar simulator (100 mW cm^−2^), with the irradiance verified using a calibrated reference cell (active area: 4 cm^2^). Forward and reverse scans were recorded at a scan rate of 10 mV s^−1^. Incident photon-to-current efficiency (IPCE) spectra were acquired using an Enlitech QE-R system over 290–850 nm. Electrochemical impedance spectroscopy (EIS) was conducted using a Zahner IM6 under dark conditions at the *V*_oc_, with a 10 mV AC perturbation over 100 mHz–1 MHz. Photoluminescence (PL) and time-resolved PL (TRPL) spectra were collected on an Edinburgh Instruments system using 472 nm pulsed-laser excitation (Edinburgh Instruments Ltd). AFM was performed in tapping mode on a Park Systems NX20 and surface parameters were extracted using XEI software. In accordance with the XEI software definitions, *R*_q_ denotes the root-mean-square (RMS) roughness and *R*_a_ the average roughness, whereas *R*_pv_ represents the peak-to-valley height difference (max–min). To capture extreme height variations in a robust manner, *R*_z_ was reported as the ten-point average roughness, defined as the arithmetic average of the five highest peaks and five lowest valleys. Contact-angle measurements of DS-modified and unmodified films were carried out using a Krüss instrument; a 2 µL droplet of DI water was used with a waiting time of 5 s. Thin film XRD analyses were performed using a Rigaku instrument. Ultraviolet visible spectroscopy (UV-vis) absorption spectra were collected using a PerkinElmer Lambda 950 spectrophotometer. Fourier transform infrared spectroscopy (FTIR) measurements were collected using a Thermo Scientific Nicolet iS50 spectrometer. A Thermo Scientific K-Alpha XPS system equipped with a monochromated Al Kα X-ray source was employed for all measurements. The analysis chamber was operated at a base pressure of ∼10^−8^ mbar. To maintain consistent sampling conditions across specimens, an X-ray spot size of 300 µm was used for every acquisition. Survey scans were recorded at 1 eV energy resolution, whereas core-level spectra were collected at 0.1 eV resolution to enable reliable discrimination of chemical states. Surface charging was mitigated using a low-energy electron flood gun for charge compensation. During data collection, the X-ray beam impinged on the sample at normal incidence (0°), and emitted photoelectrons were detected at a 45° take-off angle with respect to the analyzer axis. Thickness profiles of perovskite films were measured using a Dektak Stylus profilometer.

### Device fabrication

FTO substrates were sequentially cleaned by ultrasonication in detergent solution, deionized water, acetone, and isopropanol. After cleaning, the substrates were dried with nitrogen and treated with oxygen plasma for 10 min. The mixed SAM solution (2PACz and MeO-2PACz at 1 : 3 w/w) was prepared at 1.0 mg mL^−1^ in anhydrous isopropyl alcohol and DMF. The mixed SAM was deposited on FTO by spin coating at 5000 rpm for 30 s, followed by annealing at 100 °C for 10 min. To remove weakly bound species, the surfaces were rinsed with anhydrous isopropyl alcohol (IPA).^30^ For DS modification, DS was prepared at 0.1 mg mL^−1^ in anhydrous IPA and applied during the rinsing step. SAM coating/rinsing processes were performed in ambient air, while all subsequent deposition processes were carried out inside a glove box. A triple-cation perovskite precursor corresponding to Cs_0.05_(FA_0.9_MA_0.1_)_0.95_Pb(I_0.9_Br_0.1_)_3_ was prepared by dissolving the precursors in a DMF/DMSO mixed solvent (4 : 1 v/v). Perovskite films were deposited *via* a two-step spin-coating process (2000 rpm for 12 s, then 6000 rpm for 24 s; acceleration 500 rpm s^−1^). Anhydrous chlorobenzene anti-solvent (100 µL) was dripped at the 14th second of the second step. After deposition, films were annealed at 120 °C for 20 min to complete crystallization. PCBM was prepared at 20 mg mL^−1^ in anhydrous chlorobenzene and spin-coated on the perovskite layer at 2000 rpm for 30 s. BCP was prepared at 0.5 mg mL^−1^ in anhydrous isopropyl alcohol and spin-coated onto PCBM at 5000 rpm for 20 s. Silver (Ag) electrode was thermally evaporated under high vacuum (2 × 10^−6^ mbar) through a shadow mask (thickness: 100 nm). The device active area was fixed as 0.095 cm^2^.

## Results and discussion

**Fig. 1:**
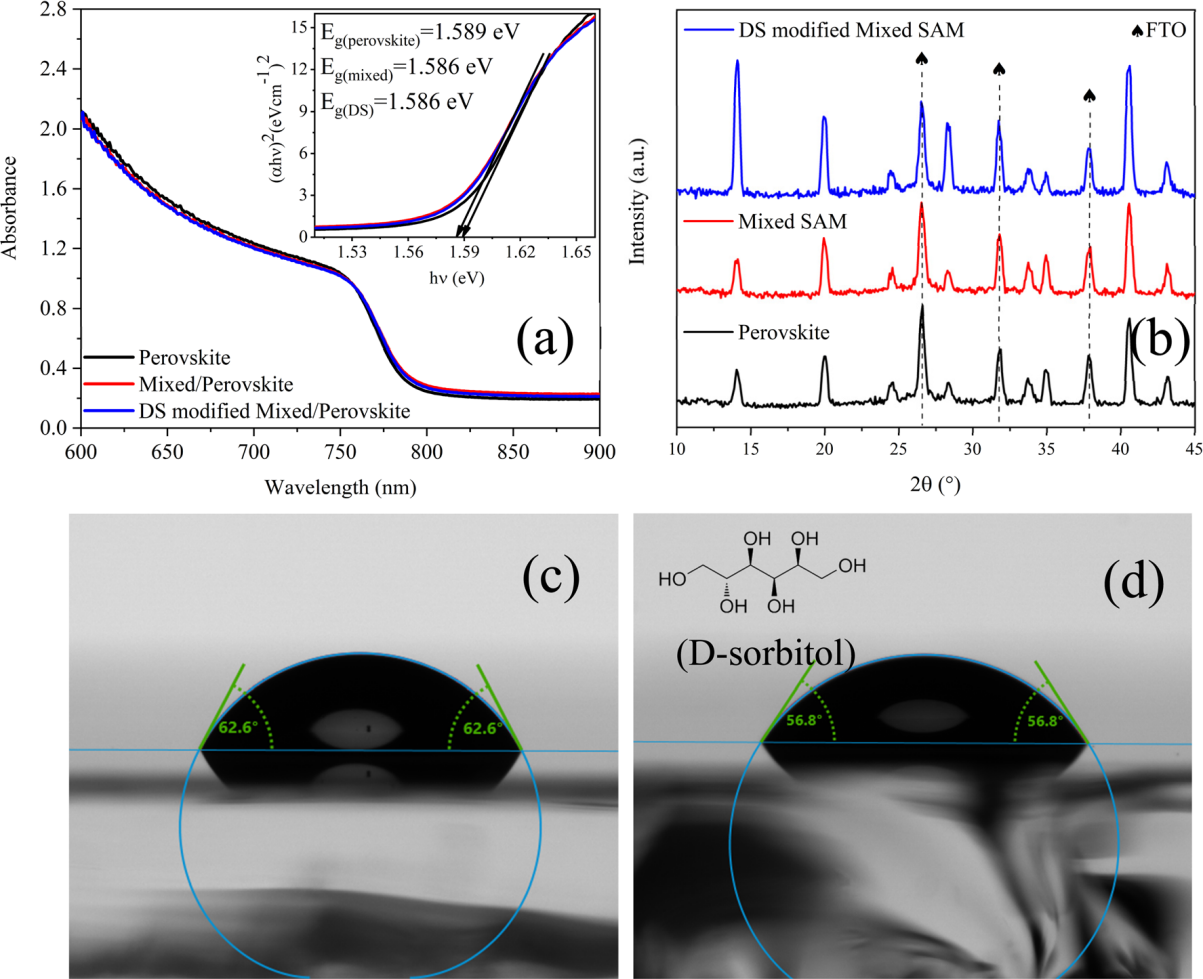
(a) UV-vis spectra of FTO/perovskite, FTO/mixed-SAM/perovskite, and FTO/DS-modified mixed-SAM/perovskite thin films (inset: Tauc plots), (b) XRD patterns of the corresponding films, (c and d) water contact-angle images of FTO/mixed-SAM and FTO/DS-modified mixed-SAM surfaces, respectively.

UV-vis absorbance spectra (Fig. 1a) show that the absorption onsets for the reference FTO/perovskite, FTO/mixed-SAM/perovskite, and FTO/DS-modified mixed-SAM/perovskite films are nearly identical, indicating that interfacial treatment does not measurably perturb the perovskite band-edge electronic structure. This is further supported by the corresponding Tauc analysis (Fig. 1a, inset), which yields nearly identical optical bandgaps (*E*_g_ = 1.589 eV for the reference and *E*_g_ = 1.586 eV for both mixed-SAM and DS-modified samples), *i.e.*, differences on the order of only a few meV and within typical fitting/experimental uncertainty.^31,32^ Notably, although the DS-modified film exhibits a slightly lower absorbance in parts of the visible range, the spectral shape and band-edge remain unchanged. Accordingly, the modest variations in absorbance amplitude across the visible range are more reasonably ascribed to extrinsic optical factors such as small differences in effective optical path length, reflectance, and roughness-induced scattering rather than to changes in the intrinsic optical transitions of the perovskite absorber.^33–36^ XRD patterns of the reference perovskite film and the films grown on the mixed-SAM and DS-modified mixed-SAM substrates are compared in Fig. 1b. All samples exhibit the characteristic perovskite reflections indexed to the (110), (200), and (220) planes at 14.1°, 19.94°, and 28.28°, respectively, confirming that neither the mixed 2PACz/MeO-2PACz interlayer nor the subsequent DS treatment measurably alters the crystalline phase or lattice structure of the perovskite.^37^ The peaks at 26.6°, 33.8°, and 37.8° are assigned to diffraction from the underlying FTO substrate.^38^ The diffraction intensities of these reflections are enhanced for the DS-modified film compared with the reference, indicating improved crystallization and/or enhanced texture induced by the modified buried interface. In conventional *θ*–2*θ* XRD, peak intensities are not solely determined by phase purity but also by thickness, crystallite coherence, and texture; accordingly, the intensity increase is therefore interpreted as consistent with improved crystallization and/or strengthened out-of-plane texture.^39^ Mechanistically, DS increases the surface polarity/wettability of the mixed-SAM, which can promote more uniform precursor spreading and modify early-stage nucleation kinetics at the buried contact, thereby facilitating more coherent grain growth and texture development.^40,41^ Given that *θ*–2*θ* peak intensities can also be affected by film thickness and surface coverage, the enhanced peak intensity is considered to be primarily consistent with improved crystallization and/or preferred texture, rather than arising from thickness differences (Fig. S1). In support of this interpretation, profilometry measurements show that the film thicknesses are comparable, with values of 540 nm for FTO/perovskite, 536.9 nm for FTO/perovskite/mixed SAM, and 523.1 nm for FTO/perovskite/DS modified mixed SAM films. Additionally, no additional diffraction features indicative of major secondary phases are observed, implying that the interfacial treatments do not induce detectable phase impurities under these processing conditions.

The effect of the mixed SAM and DS modification on the surface energy of FTO was evaluated by water contact-angle measurements. The mixed SAM surface exhibited contact angles of 62.6° and 62.6° on the left and right sides, respectively (Fig. 1c). After DS modification, the contact angle decreased to 56.8° on both sides, corresponding to an average of 56.8°. The ∼5.8° reduction indicates that DS, bearing multiple –OH groups, imparts a more polar character to the surface and improves wettability (Fig. 1d). Although this change appears modest, it can be decisive for the spreading and coating dynamics of polar perovskite precursor solvents such as DMF/DMSO; accordingly, it is expected to facilitate more uniform precursor spreading and early-stage film-formation kinetics, which can favor more homogeneous nucleation and crystallization during growth.^42–45^

**Fig. 2:**
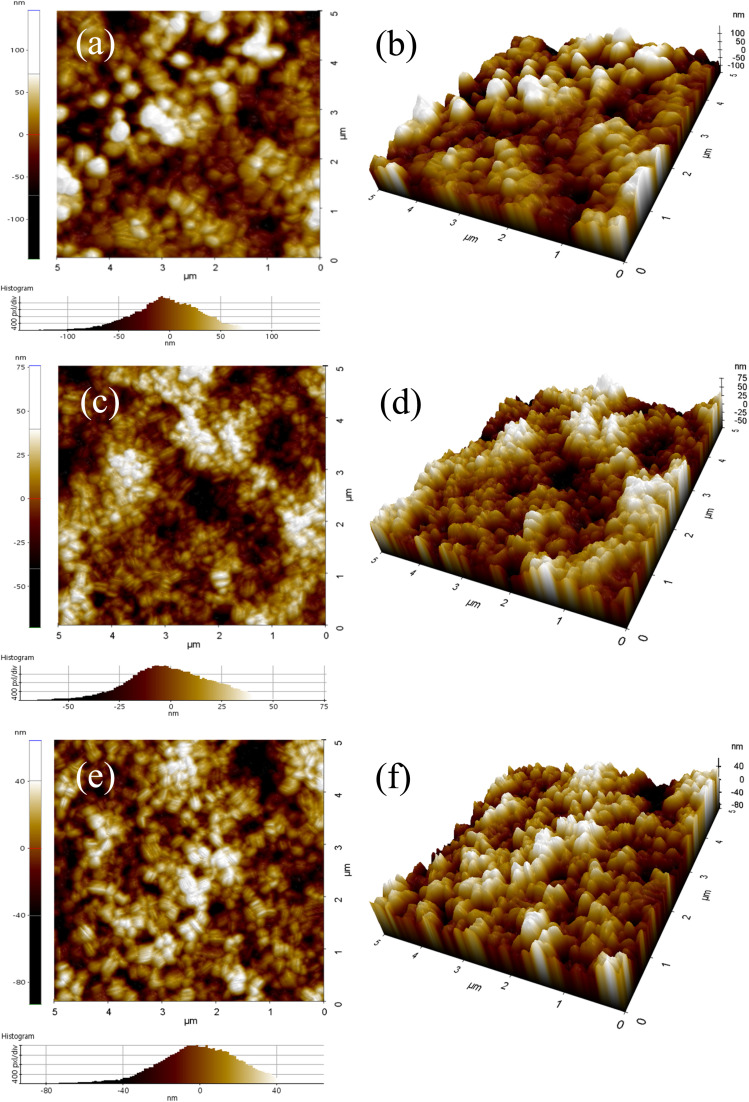
AFM topography and 3D images of (a and b) FTO/perovskite, (c and d) FTO/mixed SAM/perovskite and (e and f) FTO/DS modified mixed SAM/perovskite thin films.

AFM topography (5 × 5 µm^2^) was used to evaluate how DS modification affects the perovskite film morphology.^42^ The reference perovskite film exhibits an RMS roughness of *R*_q_ = 36.75 nm and an average roughness of *R*_a_ = 28.48 nm, whereas the perovskite grown on the mixed SAM shows markedly reduced values of *R*_q_ = 20.4 nm and *R*_a_ = 16.29 nm (Fig. 2a and c). Similarly, the DS-modified mixed-SAM sample yields *R*_q_ = 20.6 nm and *R*_a_ = 16.39 nm. In addition to lowering the average roughness, the mixed-SAM and DS-treated surfaces substantially suppress extreme height fluctuations: peak-to-valley descriptors (*R*_pv_ and *R*_z_) are high for the reference film (*R*_pv_ ≈ 294 nm; *R*_z_ ≈ 279 nm) but decrease by roughly half for the mixed-SAM and DS-modified samples (*R*_pv_ ≈ 149–158 nm; *R*_z_ ≈ 143–151 nm) (Fig. 2e). This trend is consistent with the contact-angle results, where improved precursor spreading on the mixed-SAM and DS-modified mixed-SAM surfaces is expected to mitigate local dewetting, promote more uniform nucleation/coverage during spin coating, and thereby yield a denser and more laterally homogeneous morphology. The reduction in both mean roughness (*R*_q_/*R*_a_) and peak-to-valley variations (*R*_pv_/*R*_z_) implies fewer pronounced surface protrusions and deep valleys, which can otherwise impair interlayer conformality and introduce localized electrical non-uniformities. Notably, the DS-modified mixed-SAM exhibits roughness values comparable to the mixed-SAM film, indicating that the primary reduction in perovskite surface roughness is governed by the mixed-SAM underlayer.^46–48^

Because the textured morphology of high-haze FTO can magnify interfacial reorganization, we emphasize that DS-assisted processing is particularly effective on high-haze FTO substrates, where it induces the largest changes in both topography and phase response. Accordingly, AFM topography and phase mapping were employed to systematically evaluate the substrate-dependent reorganization of the mixed-SAM induced by anhydrous IPA rinsing and DS-assisted treatment on high-haze (and, for comparison, low-haze) FTO (Table S1 and Fig. S2 and S3). On textured high-haze FTO, the non-rinsed mixed-SAM decreases the apparent roughness relative to bare FTO, consistent with partial masking of the underlying topography by a residual weakly bound organic fraction. After IPA rinsing, the topographic contrast increases markedly, indicating removal of the physisorbed overlayer and re-exposure of the textured substrate.^49^ Notably, DS-assisted rinsing yields the largest height dispersion, consistent with removal/redistribution of weakly bound organics and re-exposure of the textured high-haze FTO topography, suggesting DS-mediated reorganization/redistribution of the mixed-SAM on the textured surface. Complementary phase images support this interpretation: while bare high-haze FTO and the non-rinsed mixed-SAM exhibit substrate-like phase responses, IPA rinsing induces a pronounced phase shift and DS-assisted rinsing increases phase heterogeneity, consistent with the emergence of heterogeneous interfacial domains. In contrast, on low-haze FTO the same treatments lead to only modest changes in topography, with a slight progressive smoothing upon IPA and DS processing. Phase metrics nonetheless indicate interfacial modification upon DS treatment, while maintaining a comparatively more uniform response than on high-haze FTO substrates. Finally, scan-wide line-profile and height-distribution (histogram) analyses (avg. *x*-axis line; Fig. S4 and S5) corroborate these areal trends: the non-rinsed mixed-SAM attenuates the height modulation (topography masking) and yields a more asymmetric, non-Gaussian height distribution consistent with patchy overlayer-like accumulation, whereas IPA rinsing restores the profile amplitude and DS-assisted rinsing produces the most pronounced modulation alongside a more regularized distribution, supporting DS-mediated interfacial reorganization on the textured electrode.^50–52^

**Fig. 3:**
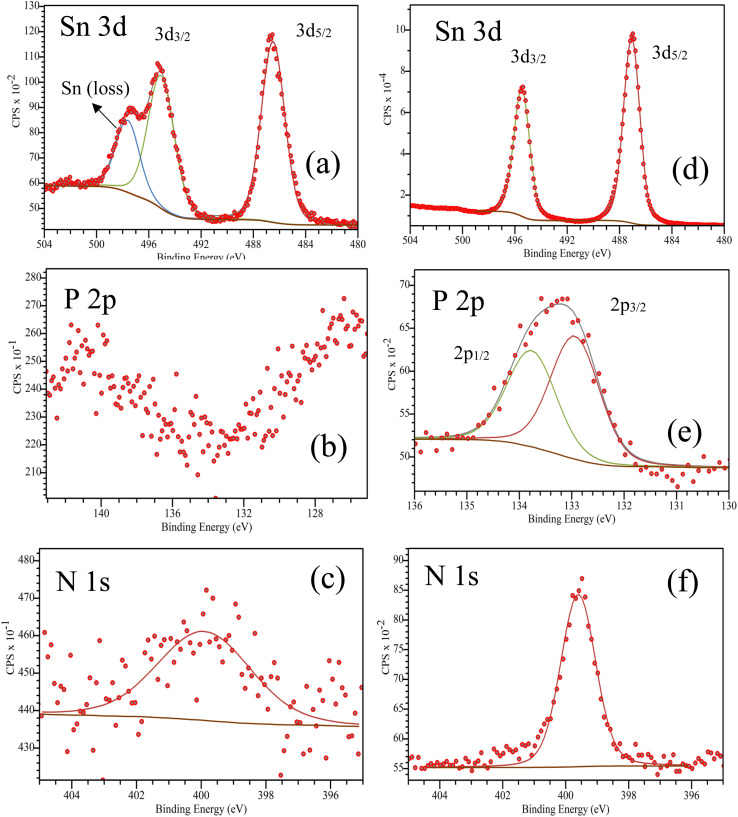
(a and d) Sn 3d, (b and e) P 2p, and (c and f) N 1s XPS core-level spectra of FTO/mixed-SAM (a–c) and FTO/DS-modified mixed-SAM films (d–f).

XPS was employed to connect the interfacial chemistry with the DS-assisted reorganization inferred from AFM analysis (Table 1). In the Sn 3d region, the IPA rinsed mixed-SAM sample is deconvoluted into Sn 3d_5/2_ and Sn 3d_3/2_ components at 486.49 eV and 495.06 eV, accompanied by a distinct high-binding-energy Sn (loss) feature centered at 497.62 eV (Fig. 3a). Importantly, both the Sn-loss signature and the P 2p signal remaining below the detection limit were reproducible across three independently probed spots on the mixed-SAM surface, indicating that these observations are not artifacts of local inhomogeneity within the analyzed areas (Fig. 3b, S4a and b). Notably, the IPA rinsed mixed-SAM sample still exhibits a weak but discernible N 1s signal (Fig. 3c), consistent with the presence of the carbazole-containing molecular overlayer, even though the corresponding phosphonate signature remains below the detection limit under the present acquisition conditions.^53,54^ In fact, under identical acquisition conditions the P 2p signal is readily detected for the same mixed-SAM deposited on low-haze FTO (Fig. S6c and d), confirming that the apparent absence on high-haze FTO arises from texture-dependent attenuation/visibility effects rather than the lack of P-containing species. This observation is consistent with the pronounced topography of high-haze FTO, which can reduce the effective XPS visibility of ultrathin headgroup signals through shadowing and local overlayer contributions within the sampling depth. Given the nanometer-scale information depth of XPS, the absence of a discernible P 2p signal in the mixed-SAM consistently observed at three different positions suggests that phosphonic headgroups are either strongly attenuated by an overlayer (*e.g.*, locally thick/aggregated organic coverage or residual adventitious carbon) and/or are not uniformly exposed within the topmost few nanometers. While DS increases the outer-surface polarity, the coupled P 2p emergence and Sn-loss suppression indicate that DS affects the interphase organization/visibility within the XPS sampling depth, consistent with a more uniform and chemically accessible anchoring configuration rather than a purely adsorbate-driven surface effect. In this respect, the concomitant appearance of a Sn energy-loss feature (∼497 eV) indicates that the underlying high haze FTO remains partially XPS-visible, consistent with incomplete/heterogeneous interfacial coverage. Notably, DS-assisted IPA processing suppresses the Sn (loss) contribution while rendering P 2p clearly detectable, pointing to a DS-driven reorganization toward a more uniform and XPS-accessible interfacial structure. After DS-assisted processing, the Sn 3d_5/2_ and Sn 3d_3/2_ peaks shift slightly to 487.07 eV and 495.47 eV, and the Sn (loss) contribution is no longer detectable, consistent with a DS-induced modification of the near-surface energy-loss signature (Fig. 3d).

Peak fitting results of the Sn 3d, P 2p and N 1s XPS spectra for mixed SAM and DS modified SAM samples

**Table:** 

Sn 3d			
Spin state	Mixed SAM	Relative area (%)	FWHM
Sn 3d_5/2_	486.49 eV	60.04	2.12 eV
Sn 3d_3/2_	495.06 eV	39.96	2.08 eV
Sn (loss)	497.62 eV	—	—

**Table:** 

Spin state	DS modified	Relative area (%)	FWHM
Sn 3d_5/2_	487.07 eV	60.01	1.47 eV
Sn 3d_3/2_	495.47 eV	39.99	1.43 eV
Sn (loss)	—	—	—

**Table:** 

P 2p			
Spin state	Mixed SAM	Relative area (%)	FWHM
P 2p_3/2_	—	—	—
P 2p_1/2_	—	—	—

**Table:** 

Spin state	DS modified	Relative area (%)	FWHM
P 2p_3/2_	132.93 eV	56.11	1.10 eV
P 2p_1/2_	133.76 eV	43.89	1.09 eV

**Table:** 

N 1s		
	Mixed SAM	DS modified
C–N/C=N	399.9 eV	399.6 eV

Consistently, the O 1s spectra evidence a restructured oxygen environment: for the IPA rinsed mixed-SAM, the lattice oxygen (Sn–O) signal appears at 530.87 eV, while a higher-binding-energy contribution at 532.09 eV is associated with surface hydroxyl/adsorbed oxygen species and oxygen in the vicinity of the phosphonate environment. Upon DS modification, the O 1s envelope can be deconvoluted into a lattice oxygen component at 530.98 eV, an interfacial oxygen contribution at 532.27 eV consistent with modified O–Sn environments and/or hydrogen-bonded oxygen at the organic/oxide interface, and an additional higher-binding-energy component at 533.04 eV, consistent with a hydroxyl-rich/polyol-related oxygen environment and/or strongly hydrogen-bonded surface oxygen species (Fig. S7a, b and Table S2). In contrast to the IPA rinsed mixed-SAM case, DS modification renders the P 2p signal clearly observable, accompanied by a more intense N 1s signal, consistent with increased effective XPS visibility and/or surface presentation of phosphonate anchoring groups after DS-assisted treatment (Fig. 3e and f).

Taken together, the concurrent suppression of the Sn (loss) feature, the evolution of O 1s components toward hydroxyl-rich/interfacial oxygen environments, and the emergence of P 2p upon DS processing support that DS does not merely rinse the surface but actively reshapes the mixed-SAM/FTO interface an effect that is most pronounced on textured, high-haze FTO, in agreement with the amplified reorganization and phase heterogeneity observed by AFM on high-haze substrates. C 1s spectra for both IPA rinsed mixed-SAM and DS-modified mixed-SAM are dominated by the main C–C/C=C component, consistent with the presence of surface-bound organic species on high haze FTO (Fig. S7c and d). A higher-binding-energy tail (∼286–289 eV) is also observed, attributable to oxygenated carbon contributions (*e.g.*, C–O and carbonyl/carboxylate species), which becomes more pronounced after DS treatment.

FTIR spectra were acquired to probe vibrational signatures associated with interfacial chemical environments in FTO/perovskite, FTO/mixed-SAM/perovskite, and FTO/DS-modified mixed-SAM/perovskite films (Fig. S8). The most pronounced spectral differences occur in the 3600–3000 cm^−1^ region, where a broad band is typically assigned to hydrogen-bonded O–H stretching arising from adsorbed moisture and/or hydrogen-bonded residual polar species, with a possible contribution from N–H stretching. Compared with the pristine FTO/perovskite reference, the mixed-SAM sample exhibits a discernible attenuation of this band, indicating modification of the surface hydroxyl/adsorbate environment upon SAM formation. Upon subsequent DS treatment, this band becomes more pronounced and broadened, consistent with an increased hydroxyl-rich and hydrogen-bonded interfacial environment at the mixed-SAM contact.^55^ Additionally, no substantial shifts are detected in characteristic perovskite vibrational modes, indicating that DS primarily alters the interfacial chemical environment rather than perturbing the bulk perovskite lattice.^56–58^

**Fig. 4:**
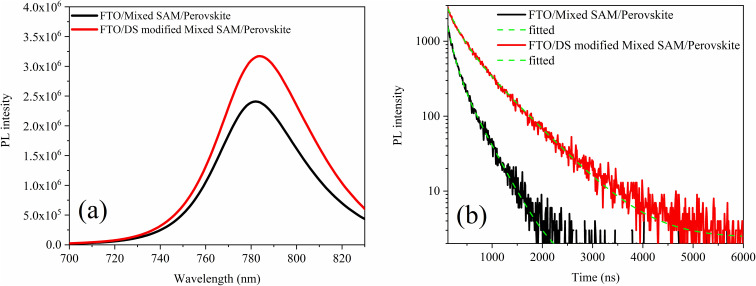
(a) PL spectra and (b) TRPL decay of FTO/mixed-SAM/perovskite, and FTO/DS-modified mixed-SAM/perovskite substrates.

To probe how the buried FTO/SAM/perovskite interface influences photocarrier recombination dynamics and non-radiative recombination losses, we performed steady-state PL and TRPL measurements on FTO/mixed-SAM/perovskite and FTO/DS-modified mixed-SAM/perovskite stacks. The steady-state PL spectra reveal a clear enhancement of the emission intensity upon DS modification, with the DS-modified mixed-SAM/perovskite exhibiting a higher PL intensity than the unmodified mixed-SAM/perovskite while maintaining a similar spectral profile (Fig. 4a). This behavior indicates that the DS modification reduces non-radiative recombination associated with the buried interface, consistent with more effective defect passivation and a lower density of interfacial trap states.^59^ The TRPL decays are well reproduced by a bi-exponential model (Fig. 4b), accounting for fast and slow decay components that are commonly assigned to distinct photophysical processes. The fast time constant (*τ*_1_) represents an early-time depopulation channel that can be influenced by interface-related charge extraction and/or fast non-radiative recombination, whereas the slow component (*τ*_2_) reflects the decay of longer-lived carriers governed by bulk and interfacial recombination processes over extended timescales.^60^ Consistent with the steady-state PL trend, both lifetime components increase upon DS modification, from *τ*_1_ = 145.91 ns and *τ*_2_ = 424.10 ns for FTO/mixed-SAM/perovskite to *τ*_1_ = 198.35 ns and *τ*_2_ = 635.05 ns for FTO/DS-modified mixed-SAM/perovskite (Table 2). The enhanced PL intensity together with the prolonged *τ*_1_ and *τ*_2_ indicate that DS modification mitigates non-radiative recombination losses and sustains a longer-lived photocarrier population at the buried FTO/mixed-SAM/perovskite interface.^59,61^

**Table 2:** TRPL fitting parameters of mixed-SAM and DS-modified mixed SAM films

Lifetime	*τ*_1_ (ns)	*τ*_1_ (ns)	*χ* ^2^
FTO/mixed-SAM/perovskite	145.91	424.10	1.297
FTO/DS-modified mixed-SAM/perovskite	198.35	635.05	1.294

**Fig. 5:**
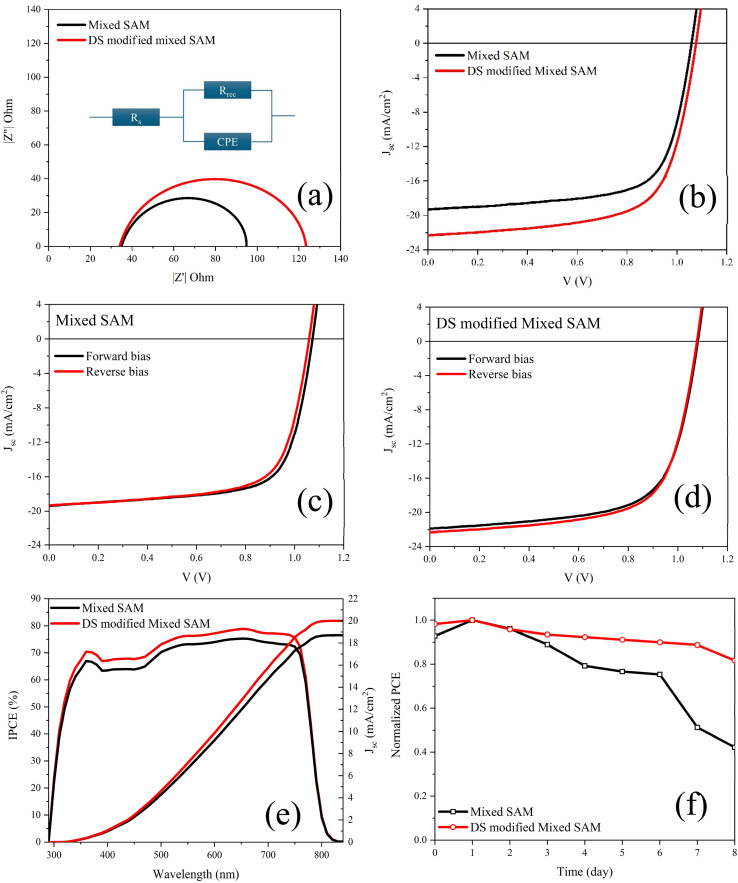
(a) Dark EIS Nyquist plots, (b) *J*–*V* characteristics, (c) forward and reverse *J*–*V* scans of the mixed-SAM device, (d) forward and reverse *J*–*V* scans of the DS-modified mixed-SAM device, (e) IPCE spectra; and (f) long-term stability of unencapsulated PSCs.

EIS was employed to elucidate how interfacial engineering influences recombination losses and the capacitive response of the devices.^62^ Impedance spectra were recorded under dark at *V*_oc_ over the 100 mHz–1 MHz frequency range, and subsequently analyzed using equivalent-circuit modeling to enable a quantitative comparison based on parameters such as the series resistance (*R*_s_) and recombination resistance (*R*_rec_) (Fig. 5a).^63,64^ For the mixed-SAM control device, *R*_s_ = 31.06 Ω, whereas the DS-modified mixed-SAM device exhibited a slightly lower value of *R*_s_ = 28.75 Ω. The recombination resistance increased markedly upon DS modification, rising from *R*_rec_ = 63.79 Ω to *R*_rec_ = 94.70 Ω. Because *R*_s_ remains nearly unchanged, the pronounced increase in *R*_rec_ points to reduced recombination under the applied bias, consistent with more effective defect passivation and/or improved contact selectivity at the interface.^65^ This electrical signature is also in line with the higher *V*_oc_ tendency observed for the DS-modified devices, since reduced interfacial recombination is expected to yield a larger effective recombination resistance.^29,66–68^

**Fig. 6:**
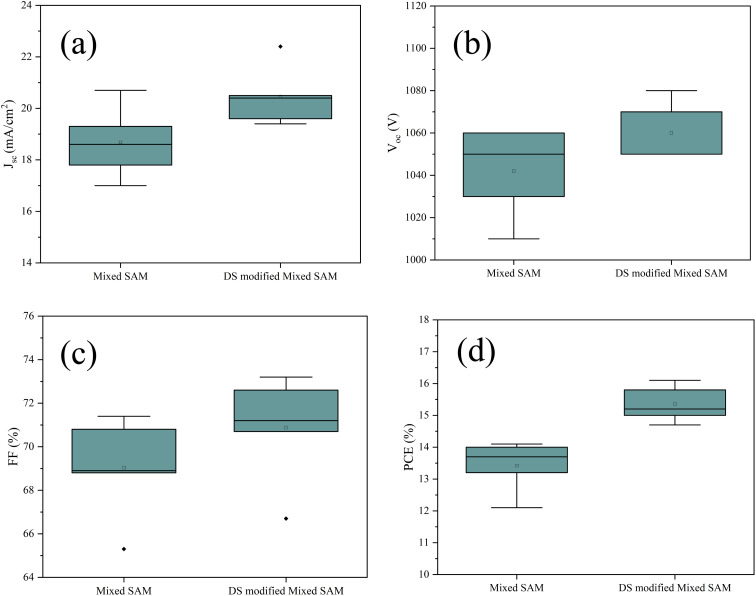
Statistical box chart of (a) *J*_sc_, (b) *V*_oc_, (c) FF and (d) PCE.

Mixed-SAM-based champion device provides, in forward/reverse scans, *J*_sc_ = 19.4/19.3 mA cm^−2^*V*_oc_ = 1070/1060 mV, FF = 70.19/68.8%, and PCE = 14.6/14.1%. The champion DS-modified mixed-SAM interface achieves higher efficiency in both scan directions, yielding *J*_sc_ = 21.9/22.4 mA cm^−2^, *V*_oc_ = 1080/1080 mV, FF = 66.6/66.7%, and PCE = 15.8/16.1% (Fig. 5b and Table 3). Although the PCE is lower than state-of-the-art p–i–n PSCs reported for fully optimized ITO-based architectures, this study intentionally employs textured high-haze FTO to elucidate buried-contact regulation under rough-TCO conditions and its impact on recombination and slow interfacial dynamics. Moreover, DS modification reduces hysteresis (Fig. 5c and d). The minimal differences in *V*_oc_ and FF between the forward and reverse scans for the DS-modified device indicate a more stable buried interface.^69,70^ This interpretation is corroborated by the dark EIS measured at the *V*_oc_. The DS-modified device exhibits an extended Nyquist arc along with an improved low-frequency impedance response, consistent with more effectively reduced recombination under operating conditions.^63,71^ Overall, DS modification preserves the morphological benefits imparted by the mixed SAM and, by regulating the recombination–ionic-process balance at the buried interface, increases *J*_sc_ and *V*_oc_ relative to the control while reducing hysteresis. The lower FF observed for the DS-modified device compared with the mixed-SAM reference together with the elimination of FF sensitivity to scan direction further suggests that the primary role of DS is not to maximize FF, but to reduce slow interfacial dynamics that otherwise degrade FF. Because FF is also influenced by transport/contact losses (*e.g.*, series resistance and interfacial extraction barriers), a modest FF trade-off in the champion device can coexist with improved recombination-limited operation and reduced dynamic losses, as evidenced by the reduced hysteresis and increased *R*_rec_.^71,72^ To clarify the respective roles of the two steps, the mixed-SAM provides the baseline hole-selective buried contact and initial interfacial regulation relative to bare FTO, whereas DS acts as a post-treatment that (i) increases surface polarity/wettability, facilitating more uniform precursor spreading and smoother perovskite growth, and (ii) reorganizes the mixed-SAM/oxide interphase on textured electrodes, as evidenced by AFM/phase mapping and the coupled XPS signatures (P 2p emergence and Sn-loss suppression). These DS-specific interfacial changes coincide with reduced hysteresis and improved recombination dynamics (higher *R*_rec_), indicating that DS primarily reduces slow interfacial dynamics at the buried contact. The IPCE spectra in Fig. 5e shows that the DS-modified mixed-SAM device exhibits higher external quantum efficiency than the mixed-SAM reference. The overall spectral profile is largely preserved, indicating that the DS treatment does not measurably alter the absorber's optical band-edge characteristics, but instead improves photocarrier generation/collection through reduced interfacial losses and consistent with improved charge collection/extraction. The integrated photocurrents derived from IPCE follow the same trend as the *J*–*V* results: for the mixed-SAM device, *J*_sc,IPCE_ = 18.7 mA cm^−2^ is close to *J*_sc,*J*–*V*_ = 19.3 mA cm^−2^, while for the DS-modified mixed-SAM device, *J*_sc,IPCE_ = 20.0 mA cm^−2^ is consistent with the higher *J*_sc,*J*–*V*_ = 22.4 mA cm^−2^. The remaining absolute differences between the two methods can be reasonably attributed to common experimental factors such as spectral calibration/irradiance mismatch, active-area definition, and the distinct excitation conditions of monochromatic IPCE *versus J*–*V* measurements. Shelf-storage stability was assessed by tracking the normalized PCE of encapsulation-free devices stored in a glovebox at ambient temperature (O_2_/H_2_O < 0.01 ppm), as shown in Fig. 5f. After a brief stabilization during the first day, the mixed-SAM reference exhibits progressive degradation, with a pronounced drop in normalized PCE after several days. In contrast, the DS-modified mixed-SAM devices show a substantially slower decay, retaining 81.7% of the initial normalized PCE after 8 days, compared to 42.2% for the mixed-SAM control. This improved inert storage stability is ascribed to DS-mediated regulation of the buried FTO/SAM/perovskite interface, which reduces non-radiative recombination losses and retards slow ionic/interfacial degradation, consistent with the reduced hysteresis, longer PL/TRPL lifetimes, and higher recombination resistance from EIS.^29,73,74^ Maximum power point tracking (MPPT) measurements were performed under continuous one-sun illumination for 600 s. Throughout the tracking period, the DS-modified mixed-SAM device maintained a consistently higher stabilized PCE than the mixed-SAM control, accompanied by a larger |*J*_mpp_|, indicating improved steady-state power output.^75^ The multi-device statistics in Fig. 6 indicate that DS modification of the mixed-SAM interface consistently improves device performance. Compared with the mixed SAM based devices, the DS-modified devices deliver a higher average photovoltaic performance, accompanied by a narrower dispersion of key metrics, most notably PCE and *V*_oc_ across the device population. This trend indicates that DS mediated buried interface engineering not only enhances the mean output but also reduces fabrication related variability, consistent with improved interfacial uniformity and reduced sensitivity to processing fluctuations. While a small number of outliers in FF are observed, the overall statistics support a more reproducible and robust performance distribution for the DS-modified interface.

**Table 3:** Champion device photovoltaic parameters of inverted PSCs incorporating mixed-SAM and DS-modified mixed-SAM (reverse scan)

Samples	*J*_sc_ (mA cm^−2^)^*J*–*V*^	*J*_sc_ (mA cm^−2^)^IPCE^	*V*_oc_ (mV)	FF (%)	PCE (%)	HI
Mixed-SAM	19.3	18.7	1060	68.8	14.1	0.032
DS modified mixed-SAM	22.4	20.0	1080	66.7	16.1	0.022

## Conclusions

In summary, we demonstrate a solution-processable buried-interface regulation strategy for inverted (p–i–n) perovskite solar cells by combining a mixed 2PACz/MeO-2PACz SAM (1 : 3, w/w) with a d-sorbitol (DS) polyol treatment. DS increases surface wettability and maintains the smooth perovskite morphology enabled by the mixed-SAM without altering the absorption edge, indicating that the performance gains originate from interfacial rather than bulk modifications. As a result, DS-modified mixed-SAM devices deliver a higher efficiency (up to 16.1% *vs.* 14.1% for the mixed-SAM control) with reduced hysteresis, consistent with reduced non-radiative recombination losses as supported by enhanced PL/TRPL responses and increased recombination resistance from EIS. Beyond performance, DS substantially improves inert shelf-storage stability under inert storage, retaining 81.7% of the initial normalized PCE after 8 days compared with 42.2% for the reference. Surface-sensitive AFM and XPS corroborate that DS-assisted processing reorganizes the mixed-SAM/FTO contact by increasing interfacial uniformity and chemical accessibility (appearance of P 2p and suppression of the Sn energy-loss signature). Collectively, these findings establish polyol-assisted mixed-SAM engineering as an effective route to mitigate slow interfacial losses and advance more stable inverted perovskite photovoltaics.

## Author contributions

Adem MUTLU: formal analysis, software, supervision, conceptualization, methodology, investigation, visualization, data curation, writing – original draft, Necip Ali TUNA – visualization, data curation, investigation, methodology, writing – original draft, Destan TOKSOZ – visualization, data curation, investigation, methodology, writing – original draft, Cem TOZLU – formal analysis, data curation, writing – original draft.

## Conflicts of interest

The authors declare no conflict of interest.

## Supplementary Material

RA-016-D6RA01990K-s001

## Data Availability

All data produced and/or analysed during this study are included in this article and its supplementary information (SI). Supplementary information: thin-film thickness data, AFM characterization of low and high-haze FTO surfaces, additional XPS spectra and peak-fitting results, FTIR spectra, and MPPT measurements supporting the findings of this study. See DOI: https://doi.org/10.1039/d6ra01990k.
