# Supplementary material for: Tuning the buried interface with d-sorbitol-modified mixed SAMs for high-efficiency inverted perovskite solar cells
Source: RSC Adv. 2026 Apr 17;16(22):20143–56. doi: 10.1039/d6ra01990k (PMC13090017; doi:10.1039/d6ra01990k)
Supplement: RA-016-D6RA01990K-s001 [file RA-016-D6RA01990K-s001.pdf]

Supplementary Information (SI) for RSC Advances.
This journal is © The Royal Society of Chemistry 2026

Supporting Information

Tuning the Buried Interface with D-Sorbitol-Modified Mixed SAMs for High-Efficiency Inverted Perovskite Solar Cells

[1,*]Adem Mutlu, [1,2]Necip Ali Tuna, [1]Destan Toksoz, [3]Cem Tozlu

[1]*Ege University, Solar Energy Institute, 35100, Izmir, Türkiye*

[2]*Department of Materials Science and Engineering, Izmir Katip Celebi University, Izmir, Türkiye*

[3]*Graphene Application and Research Center, Izmir Katip Celebi University, Cigli, 35620 Izmir, Türkiye*

***Corresponding author E-mail**: adem.mutlu@ege.edu.tr*

This file includes:

Figure S1 to S9

Table S1 to S3

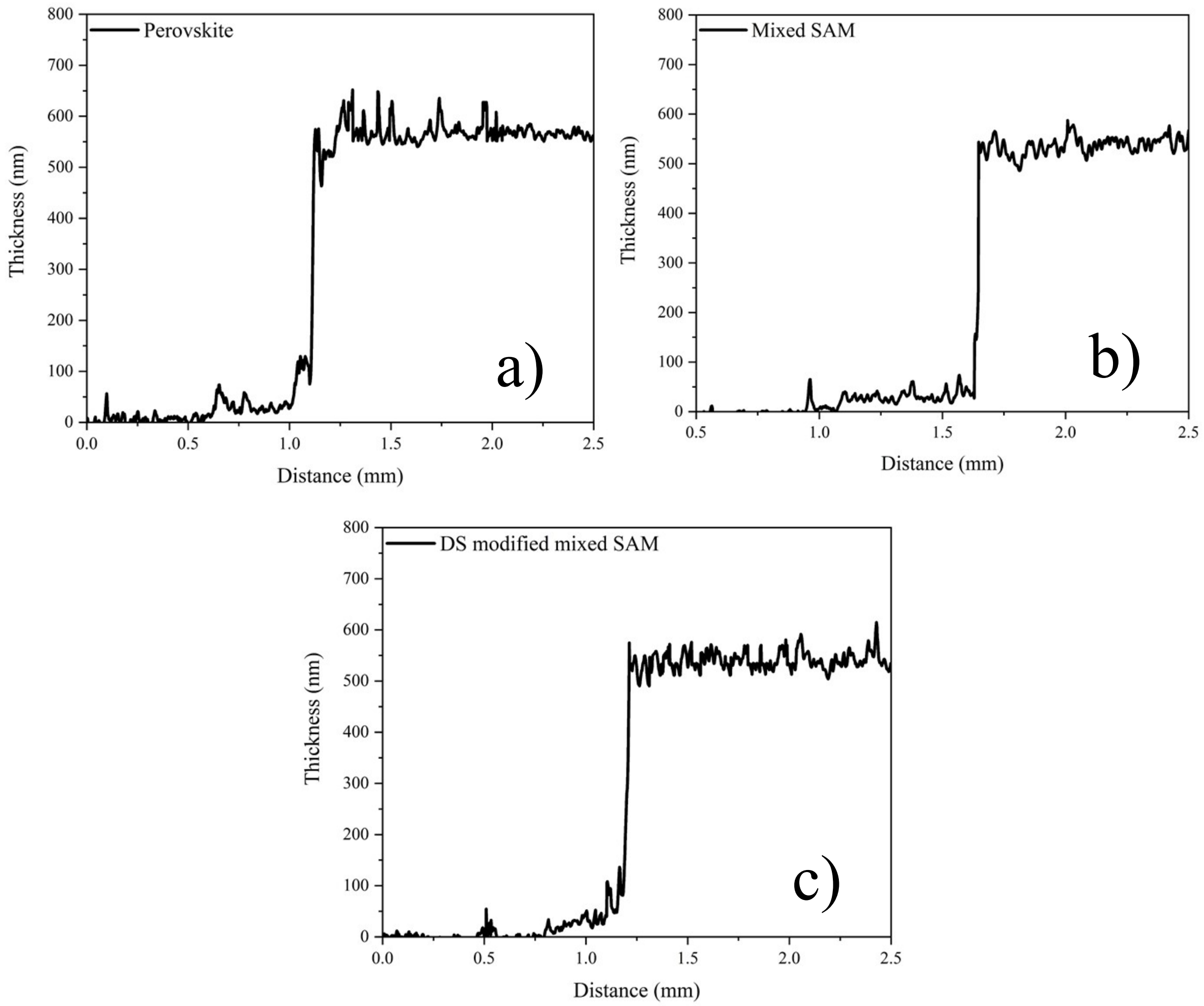

Figure S1. Stylus profilometry thickness profiles of perovskite films deposited on different buried-contact configurations: (a) FTO/perovskite, (b) FTO/Mixed-SAM/perovskite, and (c) FTO/DS-modified Mixed-SAM/perovskite.

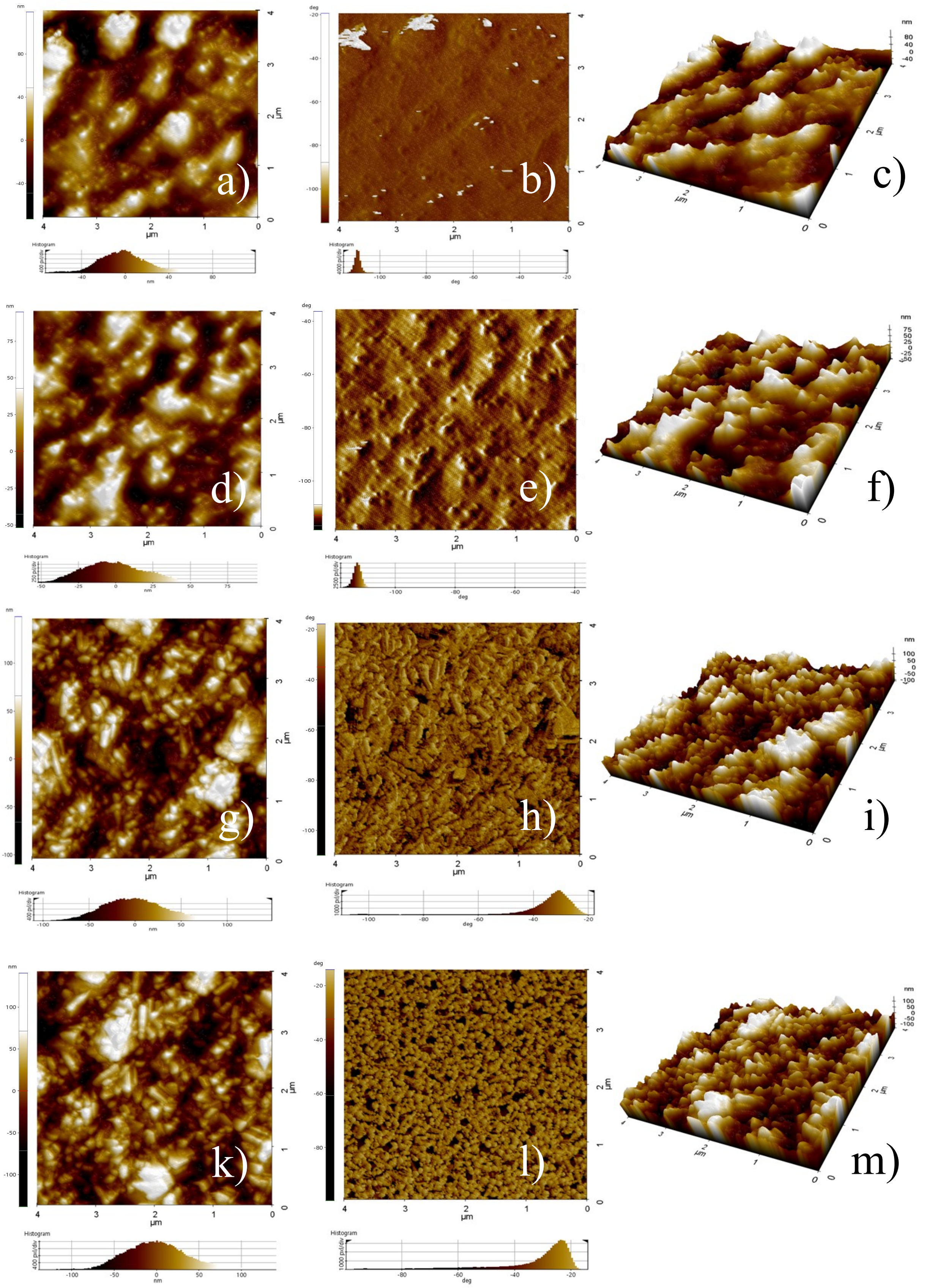

Figure S2. High-haze FTO: AFM topography, phase and 3D images of (a-c) bare FTO, (d-f) FTO/Mixed SAM (no rinse), (g-i) FTO/Mixed SAM (IPA rinsed), and (l-m) FTO/DS modified mixed SAM (DS in IPA) thin films.

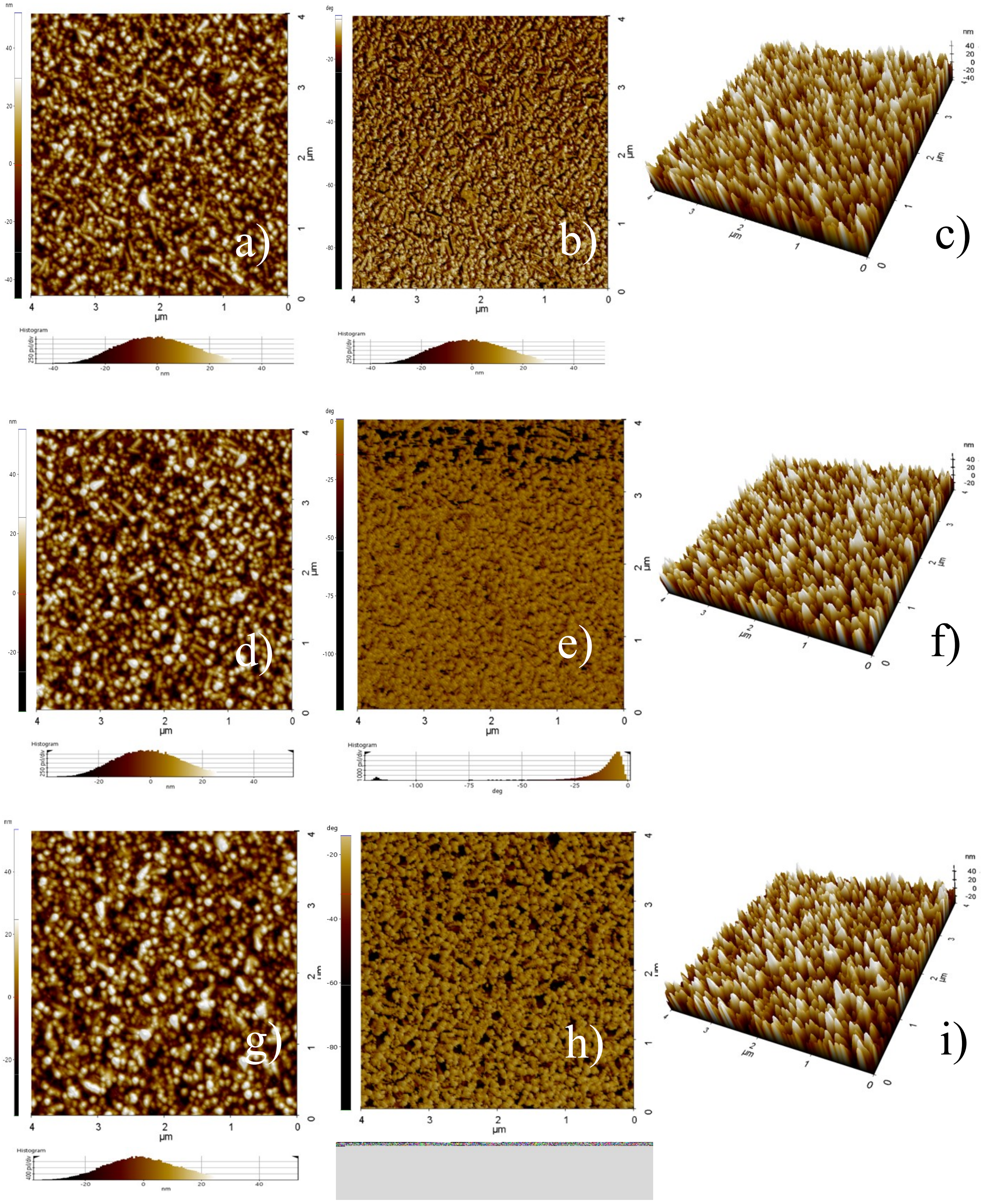

Figure S3. Low-haze FTO: AFM topography, phase and 3D images of (a-c) bare FTO, (d-f) FTO/Mixed SAM (IPA rinsed) and (g-i) FTO/DS modified mixed SAM (DS in IPA) thin films.

Table S1. AFM topography and phase metrics for mixed-SAM and DS-modified mixed-SAM on low and high-haze FTO (scan area: 4 × 4 µm^2).

**Substrate / treatment**	**Topography R_q (nm)**	**Topography R_{pv} (nm)**	**Phase mean (deg)**	**Phase R_q (deg)**
**High-haze FTO (bare)**	24.905	191.776	−108.084	10.324
**High-haze FTO/mixed-SAM (no IPA rinse)**	21.796	146.621	−112.745	2.056
**High-haze FTO/mixed-SAM (IPA-rinsed)**	33.623	258.636	−34.849	12.018
**High-haze FTO/DS-modified mixed-SAM (DS in IPA rinse)**	36.559	279.325	−52.100	20.848
**Low-haze FTO (bare)**	14.092	98.633	−15.836	4.311
**Low-haze FTO /mixed-SAM (IPA-rinsed)**	13.200	95.180	−14.154	21.218
**Low-haze FTO /DS-modified mixed-SAM (DS in IPA rinse)**	12.591	91.322	−32.348	14.473

R_q: RMS roughness; R_{pv}: peak-to-valley height range. Phase values are extracted from NCM phase backward images.

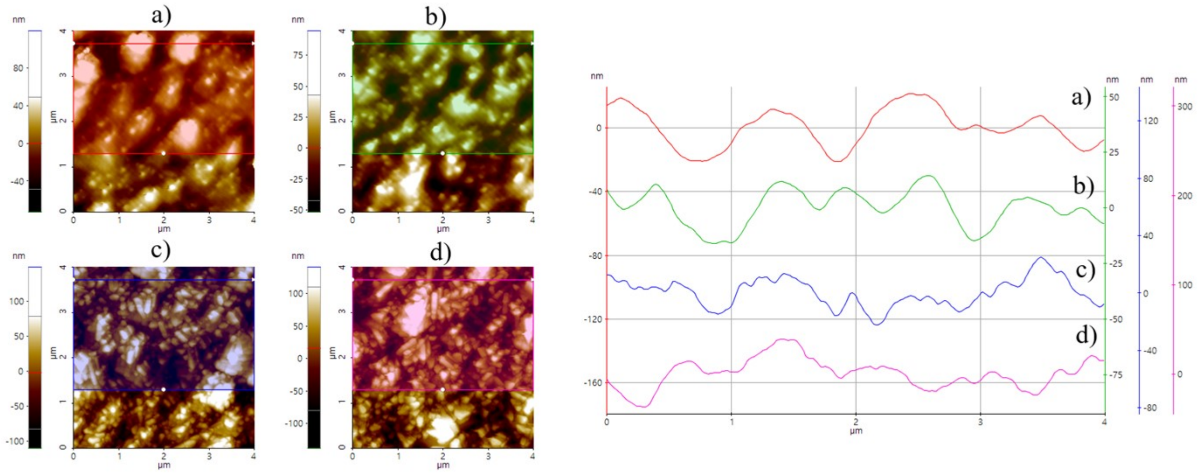

Figure S4. Representative AFM height maps (4 × 4 µm^2) and corresponding line profiles extracted along the same horizontal scan line (colored line) for high-haze FTO before and after mixed-SAM processing: (a) bare high-haze FTO, (b) FTO/mixed-SAM (non-rinsed), (c)

FTO/mixed-SAM after rinsing with IPA, and (d) FTO/DS-modified mixed-SAM after rinsing with DS-containing IPA.

a)

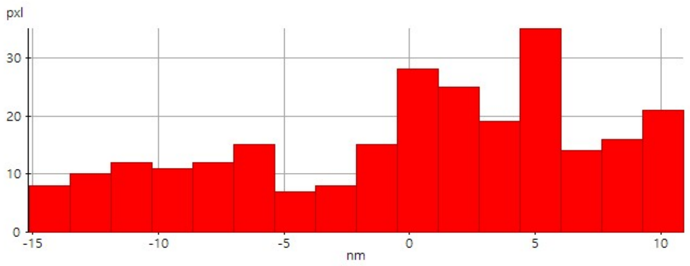

Statistics

Line	Min(nm)	Max(nm)	Mid(nm)	Mean(nm)	Rpv(nm)	Rq(nm)	Ra(nm)	Rz(nm)	Rsk	Rku
Red	-15.182	10.872	-2.155	0.000	26.055	6.992	5.828	18.664	0.476	2.166

b)

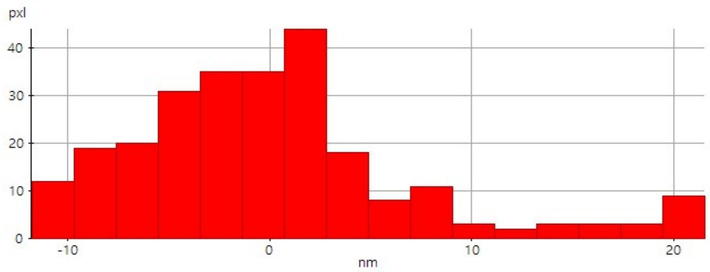

Statistics

Line	Min(nm)	Max(nm)	Mid(nm)	Mean(nm)	Rpv(nm)	Rq(nm)	Ra(nm)	Rz(nm)	Rsk	Rku
Red	-11.806	21.497	4.846	0.000	33.303	7.114	5.210	14.961	-1.081	4.375

c)

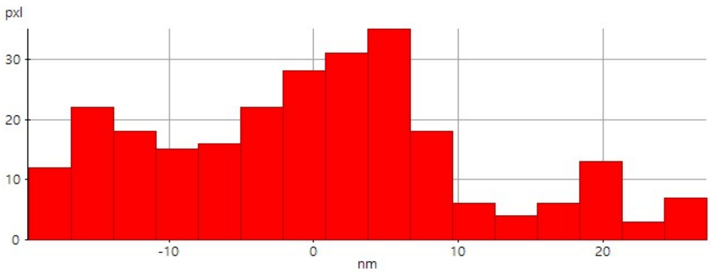

Statistics

Line	Min(nm)	Max(nm)	Mid(nm)	Mean(nm)	Rpv(nm)	Rq(nm)	Ra(nm)	Rz(nm)	Rsk	Rku
Red	-19.765	27.135	3.685	0.000	46.900	11.041	8.781	33.720	-0.334	2.656

d)

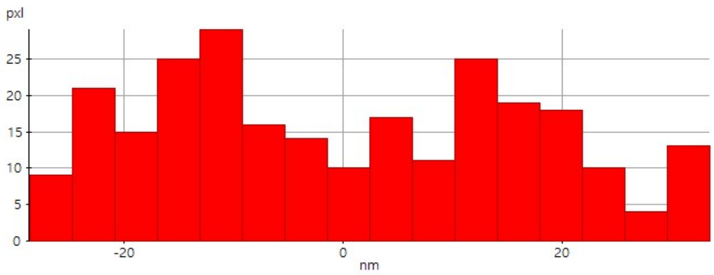

Statistics

Line	Min(nm)	Max(nm)	Mid(nm)	Mean(nm)	Rpv(nm)	Rq(nm)	Ra(nm)	Rz(nm)	Rsk	Rku
Red	-28.660	33.398	2.369	0.000	62.058	16.823	14.777	44.699	-0.199	1.904

Figure S5. Scan-wide height-distribution (histogram) analysis derived from the 4×4 μm^2 AFM height maps for high-haze FTO surfaces: (a) bare high-haze FTO, (b) FTO/mixed-SAM (non-

rinsed), (c) FTO/mixed-SAM after rinsing with IPA, and (d) FTO/DS-modified mixed-SAM after rinsing with DS-containing IPA.

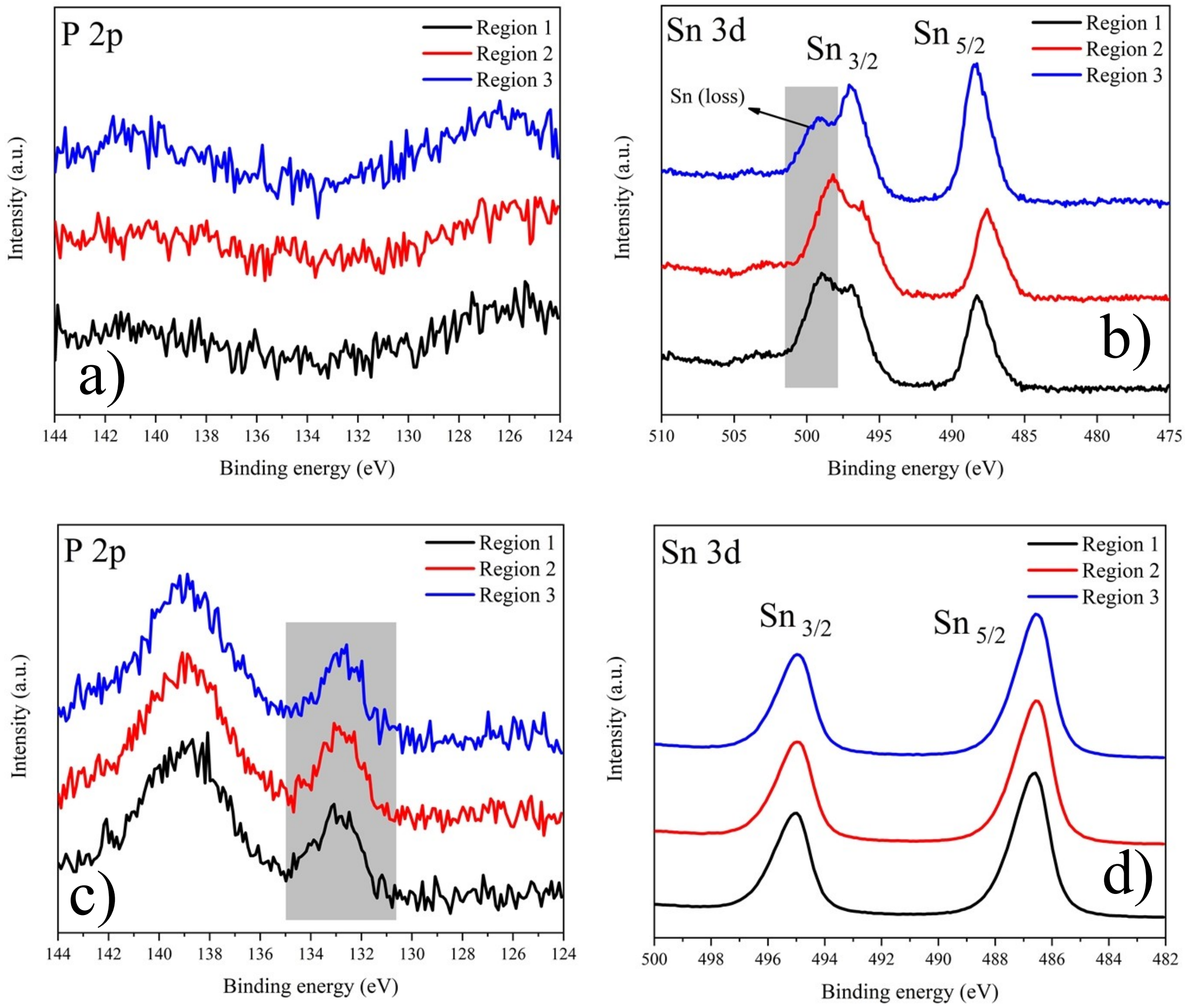

Figure S6. High-resolution a) P 2p and b) Sn 3d XPS spectra collected from three different spots (Region 1–3) on high-haze FTO/mixed-SAM (IPA rinsed) substrate. High-resolution c) P 2p and d) Sn 3d XPS spectra collected from three different spots (Region 1–3) on low-haze FTO/mixed-SAM (IPA rinsed) substrate.

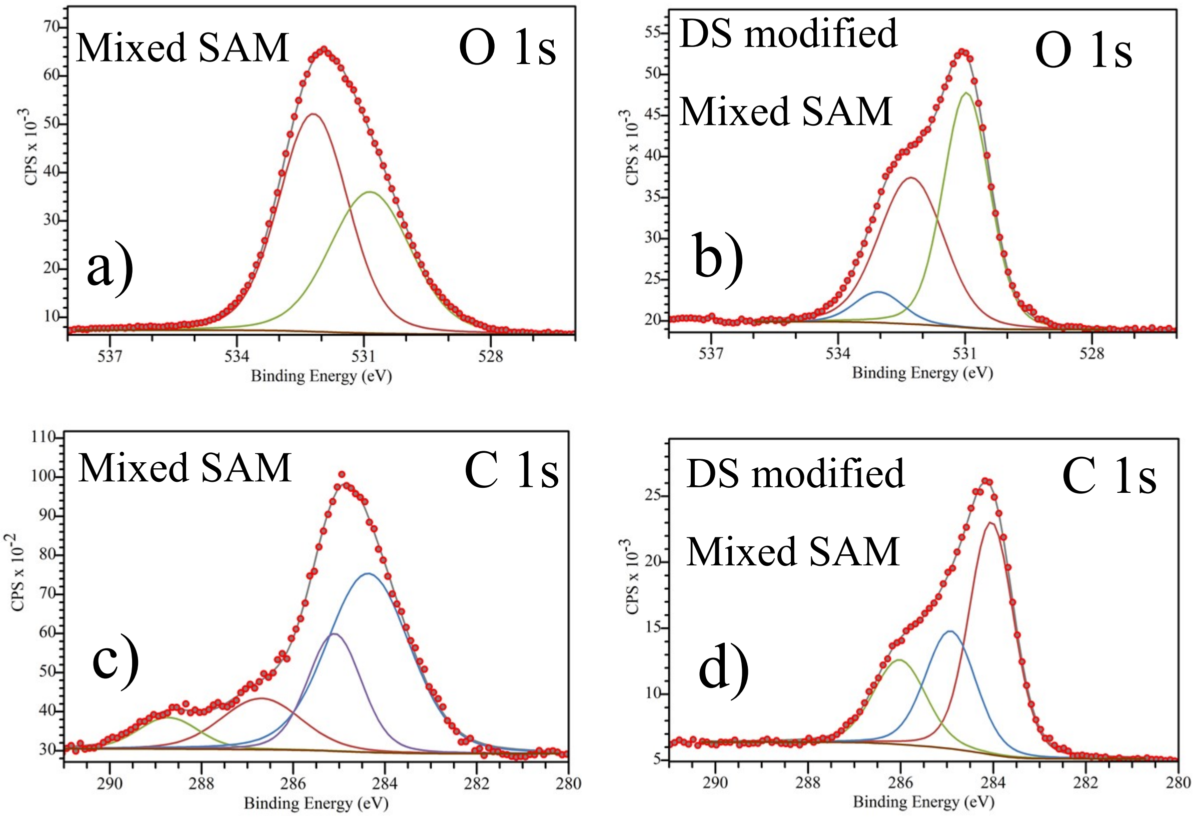

Figure S7. (a, b) O 1s and (c, d) C 1s XPS core-level spectra of (a) FTO/mixed-SAM and FTO/DS-modified mixed-SAM films.

Table S2. Peak fitting results of the O 1s XPS spectra for mixed SAM and DS modified SAM samples.

**O 1s**	**Mixed SAM**	**Relative area (%)**	**FWHM (eV)**
lattice O^{2-} (Sn–O)	530.87 eV	43.08	2.24
Surface –OH / adsorbed H_2O/O_x species	532.09 eV	56.92	1.92
**O 1s**	**DS modified**	**Relative area (%)**	**FWHM (eV)**
lattice O^{2-} (Sn–O)	530.98 eV	49.93	1.30
interfacial O (P–O–Sn)/surface –OH/adsorbates	532.27 eV	43.29	1.80
hydroxyl-rich oxygen (polyol-related and/or strongly H-bonded	533.04 eV	6.78	1.35

adsorbates			

Table S3. Peak fitting results of the C 1s XPS spectra for mixed SAM and DS modified SAM samples.

**C 1s**	**Mixed SAM**	**Relative area (%)**	**FWHM (eV)**
C–C / C=C (hydrocarbon/aromatic)	284.36	54.61	2.12
C–N and/or C–O (heteroatom-adjacent carbon)	285.10	23.27	1.38
C–O (ether/alcohol; methoxy-rich and/or H-bonded C–O)	286.70	15.13	2.05
Carbonate/carboxylate-like surface adsorbates; possible adventitious carbon	288.72	6.99	1.54
**C 1s**	**DS modified**	**Relative area (%)**	**FWHM (eV)**
C–C / C=C (hydrocarbon/aromatic)	284.14	44.86	1.14
C–C/C–H shoulder + heteroatom-adjacent C (C–N/C–O overlap)	284.91	35.64	1.54
Oxygenated carbon (C–O-dominant; alcohol/ether/polyol/methoxy environments; possible H-bonded C–O)	286.09	19.50	1.41

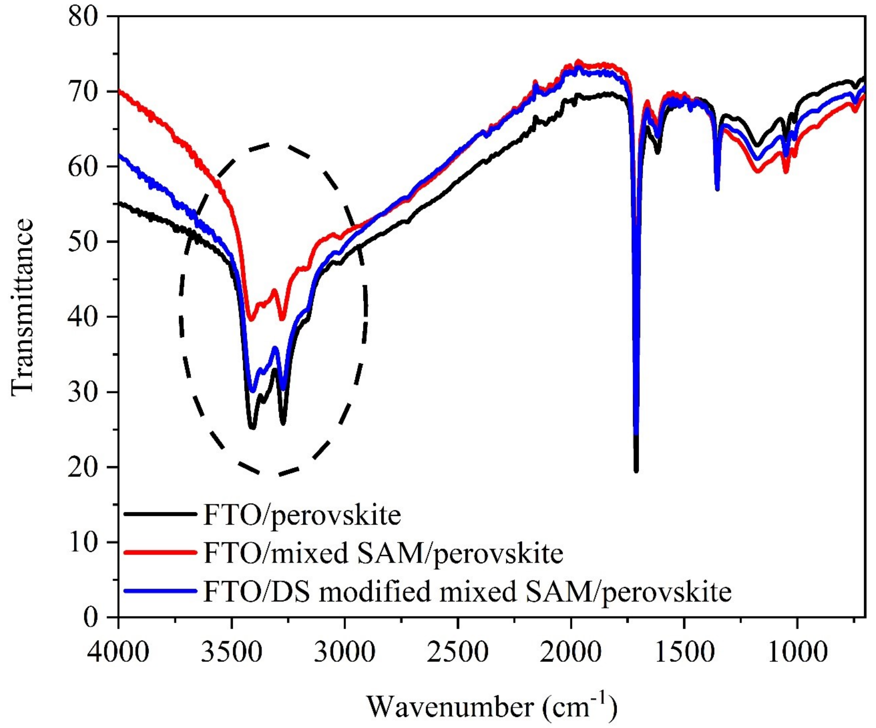

Figure S8. FTIR spectra of FTO/perovskite, FTO/mixed-SAM/perovskite, and FTO/DS-

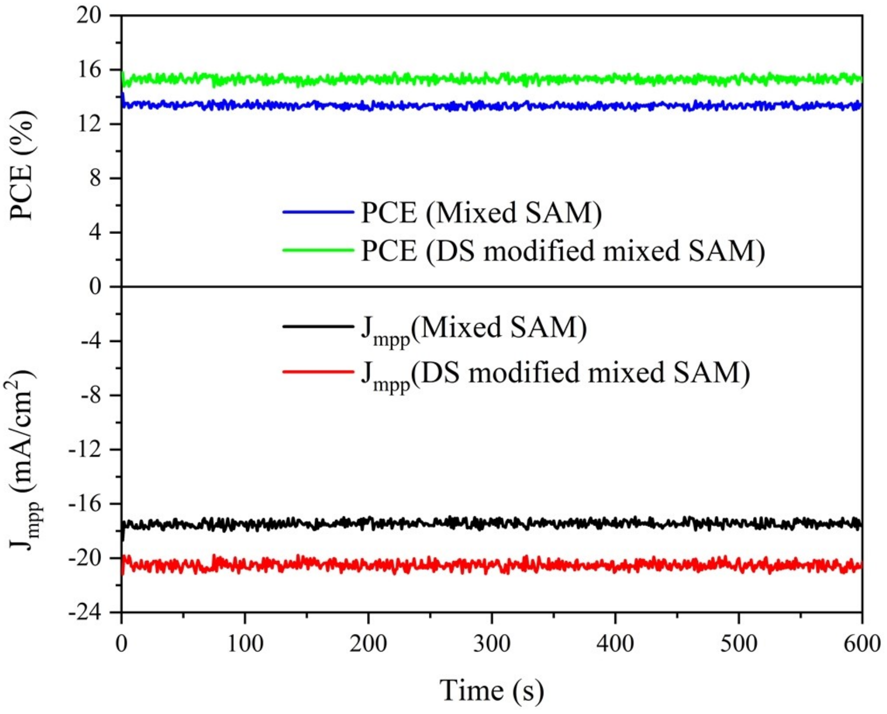

modified mixed-SAM/perovskite films.

Figure S9. Time-dependent maximum power point tracking of mixed-SAM and DS-modified mixed-SAM based PSCs measured for 600 s under continuous illumination. (The MPPT measurements were performed on an independently fabricated set of devices prepared under the same optimized conditions as the representative devices in the main text.)
